# Oral route lipopolysaccharide as a potential dementia preventive agent inducing neuroprotective microglia

**DOI:** 10.3389/fimmu.2023.1110583

**Published:** 2023-03-09

**Authors:** Haruka Mizobuchi

**Affiliations:** Laboratory of Molecular Immunology, Department of Animal Resource Sciences, Graduate School of Agricultural and Life Sciences, The University of Tokyo, Tokyo, Japan

**Keywords:** microglia, lipopolysaccharide, oral administration, neuroprotection, cognitive dysfunction, dementia

## Abstract

In today’s aging society, dementia is an urgent problem to be solved because no treatment or preventive methods have been established. This review focuses on oral administration of lipopolysaccharide (LPS), an outer membrane component of Gram-negative bacteria, as a novel preventive drug for dementia. LPS is also called endotoxin and is well known to induce inflammation when administered systemically. On the other hand, although we humans routinely ingest LPS derived from symbiotic bacteria of edible plants, the effect of oral administration of LPS has hardly been studied. Recently, oral administration of LPS was reported to prevent dementia by inducing neuroprotective microglia. Furthermore, it has been suggested that colony stimulating factor 1 (CSF1) is involved in the dementia prevention mechanism by oral administration of LPS. Thus, in this review, we summarized the previous studies of oral administration of LPS and discussed the predicted dementia prevention mechanism. In addition, we showed the potential of oral LPS administration as a preventive drug for dementia by highlighting research gaps and future issues for clinical application development.

## Introduction

Currently, as the aging of the population progresses worldwide, dementia such as Alzheimer’s disease caused by aging is one of the global issues to be resolved. The WHO reports that there are currently over 55 million people with dementia, and this number is expected to reach 75 million in 2030 and 132 million in 2050 (1). However, there is still no effective preventive or therapeutic method for dementia, and urgent research and development is required. As research progresses, it has become clear that the activation of microglia, which are tissue-resident macrophages in the central nervous system (CNS), is important in the pathology of dementia. Microglia, which play a central role in the innate immune system, control neuronal activity through synaptic phagocytosis and cytokine production in the physiological condition and maintain CNS homeostasis. On the other hand, pathological microglia, which are activated and induced in pathological conditions, contribute to neural network damage through xenophagocytosis, inflammatory factors, and cytotoxic factors (2). Therefore, controlling microglial activation can lead to the prevention and treatment of dementia.

Lipopolysaccharide (LPS) is a glycolipid that constitutes the outer membrane of Gram-negative bacteria. LPS activates various cells including macrophages *via* toll-like receptor 4 (TLR4). LPS, also called endotoxin, is well known to cause fever, systemic inflammation, and shock when injected intravenously (The LD50 for intraperitoneally administered LPS is 27 mg/kg.) (3). In the field of neuroscience, a single systemic administration of LPS at a high dose (approximately 5 mg/kg) has been generally used as a Parkinson’s model or an encephalitis model to induce neuroinflammation (4, 5). It has been reported that a single high-dose LPS systemic administration promotes amyloid-β accumulation and exacerbates cognitive decline in Alzheimer’s disease models (6). The underlying mechanism of this neuropathy involves inflammatory microglia induced by a single high dose of LPS (7). On the other hand, repetitive low-dose systemic administration of LPS (0.2-0.9 mg/kg) contributes to the prevention of neurological dysfunctions such as Alzheimer’s disease and ischemic neuropathy by inducing neuroprotective microglia (8, 9). The neuroprotective effects of repetitive low-dose LPS administration have already been described in detail in our previous review (10).

However, even with repeated low doses of LPS, systemic administration of LPS causes body weight loss in experimental animals (8). Therefore, the problem is that systemic administration of LPS and subsequent elevation of blood LPS concentration (bioavailability), even in very small doses, carry the risk of systemic inflammation. In contrast, we have elucidated that oral administration of LPS (OAL) at a dose of 0.01-1 mg/kg contributes to the prevention and treatment of various diseases while maintaining high safety (11, 12). Furthermore, we have recently shown that OAL prevents dementia by inducing neuroprotective microglia (13, 14). In this review, we summarize previous studies on OAL and propose the potential of OAL as a new therapeutic strategy. The data sources for this review were comprehensively searched using the PubMed with keywords of “lipopolysaccharide” and “oral administration/treatment” (last searched February 16, 2023). Data published within the last ten years were prioritized, focusing on the novel and comprehensive reports.

### Distinct mechanism between oral and intravenous administration of LPS

LPS is a glycolipid that constitutes the outer membrane of Gram-negative bacteria, and induces strong fever, systemic inflammation, and shock when administered intravenously (3). Therefore, LPS is called endotoxin, and intravenous administration of LPS has long been used as a model of sepsis and systemic inflammation. The maximum tolerated dose of LPS for intravenous administration in humans is 4 ng/kg, and the inclusion of LPS in injection drug is prohibited (15). Under these circumstances, LPS has been recognized as a dangerous substance that should be eliminated in the medical field. On the other hand, a minority research group including ours has found that LPS is contained in many edible plants and that human beings take LPS orally on a daily basis. Furthermore, it has been elucidated that orally administered LPS suppresses rather than promotes inflammation and contributes to disease prevention. In other words, LPS exhibits completely opposite effects when administered orally and when administered intravenously. Now, the “aufheben” of recognition for LPS is about to occur from endotoxin as an inflammatory inducer to disease preventive agent *via* oral route.

In fact, LPS is detected in many edible plants, including wheat, rice, and buckwheat (16). The LPS is derived from symbiotic bacteria of edible plants. Other Gram-negative bacteria found in edibles include *Acetobacter* spp. used to produce vinegar and Caspian Sea yogurt, *Zymomonas* spp. and *Xanthomonas* spp. used to produce tequila, nata de coco and xanthan gum, and *Enterobacter* spp. and *Pantoea* spp. used to ferment salapao in Thailand and rye bread in Finland (16). In other words, we humans have already unconsciously orally ingested LPS derived from food. In fact, animal safety studies have confirmed that LPS is not toxic when administered orally, even at doses of 500-4500 mg/kg, which are far higher than doses that are toxic when administered intravenously (16–18). Therefore, it is thought that the pharmacokinetics of LPS are completely different between intravenous and oral administration, resulting in different pharmacological effects.

### Studies on disease prevention effect of OAL

Previous studies on OAL are summarized in **Table 1**. Although there are very few OAL studies compared to studies in the LPS intravenous model, most of them demonstrate health maintenance effects of OAL. Märklin et al. revealed that OAL regulates granulopoiesis by inducing hematopoietic stem cells and progenitor cells into the neutrophil lineage *via* TLR4 (19). This study indicates that OAL contributes to maintaining homeostasis.

**Table 1:** Studies on oral administration of LPS.

Origin of LPS	Dose	Animal	Results	References
*P. agglomerans*	10-1,000 mg/kg for 28 days	Rat	Safety testing of OAL	Taniguchi et al., 2009 (16)
*P. agglomerans*	500, 1500, or 4500 mg/kg/day for at least 90 days.	Rat	Safety testing of OAL	Phipps et al., 2020 (17)
*E. coli* (O111:B4)	12 μg/kg, once	Calf	Safety testing of OAL	Samarasinghe et al., 2020 (18)
*E. coli* (JM83)	5 mg/L drinking water for 11 days	Mouse	OAL regulates TLR4-dependent granulopoiesis	Märklin et al., 2020 (19)
*E. coli* (O127:B8)	1-4 μg/μl drinking water for 7 days	Mouse	OAL enhances the innate immune resistance against vancomycin-resistant Enterococcus.	Brandl et al., 2008 (20)
*E. coli* (O111:B4)	25 mg/kg three times per week for 3 weeks	Mouse	OAL reverses the attenuation of cancer immunotherapy effects caused by antibiotics.	Iida et al., 2013 (21)
*E. coli* (O111:B4)	50 μg/ml drinking water for 4 weeks	Mouse	OAL prevents antibiotic-induced reduction of intestinal Csf1 and intestinal macrophages.	Muller et al., 2014 (22)
*E. coli* (O127:B8)	1 mg/feed at intervals of 2-3 days for five times	Rat	OAL in combination with myelin basic protein suppresses experimental autoimmune encephalomyelitis.	Khoury et al., 1990 (23)
*E. coli* (O55:B5)	50 μg/100 μl drinking water every 4 days for five times	Mouse	OAL protects against sepsis induced by cecal ligation and puncture.	Márquez-Velasco et al., 2007 (24)
*E. coli* (O111:B4)	0.01, 0.05, or 0.1 μg/kg during week-2, -1, and +1 relative to parturition.	Cow	OAL increases plasma anti-LPS IgM antibodies.	Ametaj et al., 2012 (25)
*E. coli* (O111:B4)	(i) 0.01 μg/kg on day-14 and -10, (ii) 0.05 μg/kg on day-7 and -3, and (iii) 0.1 μg/kg on day3 and 7 relative to parturition	Cow	OAL modulate the profile of plasma metabolites and minerals postpartum.	Zebeli et al., 2012 (26)
*E. coli* (O111:B4)	(i) 0.01 μg/kg once on day –28; (ii) 0.05 μg/kg twice on day–25 and –21; (iii) 0.1 μg/kg twice on d ay–18 and –14 relative to parturition	Cow	OAL increases IgA antibodies in saliva.	Iqbal et al., 2014 (27, 28)
*E. coli* (O127:B8)	2-4 μg/mouse every other day from birth till 4 weeks of age	Mouse	OAL induces local immunological changes in the pancreatic lymph nodes.	Kihl et al., 2019 (29)
*P. agglomerans*	10 μg/kg for 35 days	Chicken	OAL enhances the effect of anticancer drugs.	Hebishima et al., 2010 (30)
*P. agglomerans*	0.5 or 1 mg/kg for 60 days	Mouse	OAL enhances the effect of anticancer drugs	Hebishima et al., 2011 (11)
*P. agglomerans*	0.68 or 6.8 μg/kg for 31 days	Mouse	OAL improves atopic dermatitis.	Wakame et al., 2015 (31)
*P. agglomerans*	0.3 or 1 mg/kg for 16 weeks	Mouse	OAL prevents arteriosclerosis.	Kobayashi et al., 2018 (12)
*P. agglomerans*	1 mg/kg for 5-19 weeks	Mouse	OAL prevents dementia via microglia.	Kobayashi et al., 2018 (13) Mizobuchi et al., 2021 (14)
*P. agglomerans*	0.2 mg/day for 60 days	Human	OAL reduces fasting blood glucose and LDL.	Nakata et al., 2011 (32)
*P. agglomerans*	0.6 mg/day for 3 months	Human	OAL suppresses bone density loss in middle-aged and elderly women.	Nakata et al., 2014 (33)
*P. agglomerans*	0.4 mg/day for 3 months	Human	OAL improves bloodstream.	Nakata et al., 2018 (34)
*E. coli* (O111:B4)	1 mg/mouse on days 50 and 80	Mouse	OAL exacerbates collagen-induced arthritis.	Yoshino et al., 1999 (35)
*S. minnesota* (T4270) and E. coli (O26:B6)	10, 100, or 200 μg/mouse (injected into the gut)	Mouse	OAL induces autoimmune symptoms in the autoantibody transgenic mice.	Murakami et al., 1994 (36)
*E. coli* (O55:B5)	2 mg/kg, once	Chicken	OAL exacerbates heat stress-induced enteritis.	Reisinger et al., 2020 (37)

It has also been reported that OAL has the effect of reducing the side effects of antibiotics. Antibiotics significantly reduce intestinal vancomycin-resistant killing by inducing downregulation of the C-type lectin RegIIIγ, whereas Brandl et al. show that OAL re-induces RegIIIγ to enhance innate immunity against vancomycin-resistant bacteria (20). In addition, although antibiotics reduce the TNF-induced effects of cancer immunotherapy, Iida et al. demonstrated that OAL restores TNF expression and TNF-producing leukocytes in tumors (21). Muller et al. demonstrated that antibiotics inhibit colony stimulating factor 1 (CSF1)-mediated gastrointestinal motility regulation by crosstalk between macrophages and intestinal neurons, while OAL prevents antibiotic-induced intestinal CSF1 downregulation and intestinal macrophage depletion (22).

Furthermore, disease preventive effects of OAL have also been reported. Khoury et al. showed that OAL in combination with myelin basic protein (MBP) suppressed experimental autoimmune encephalomyelitis (EAE) (23). Márquez-Velasco et al. revealed that OAL protects against sepsis induced by cecal ligation and puncture (24). Ametaj et al. reported that OAL to perinatal dairy cows modulates the postpartum plasma metabolite and mineral profiles and enhances the mucosal IgA (25–28). Kihl et al. showed that OAL induced suppression of inflammatory cytokine expression in pancreatic lymph nodes (29). These studies indicate that OAL is effective in maintaining homeostasis and in preventing or treating disease. Incidentally, all the LPS used in the above studies are derived from *Escherichia coli*.

*Pantoea agglomerans* is one of the plant symbiotic bacteria, and widely exists in edible plants such as wheat, rice, and root vegetables. *P. agglomerans* promotes the growth of symbiotic plants by suppressing fungal growth, fixing nitrogen, and dissolving inorganic phosphorus (38). In fact, the bacteria are also used as a food preservative (39, 40). In addition, the safety of oral administration of *P. agglomerans*-derived LPS is guaranteed by objective tests based on Organization for Economic Co-operation and Development (OECD) standards (17). Therefore, we focused on this *P. agglomerans*-derived LPS as a food-experienced LPS, and elucidated the preventive and therapeutic effects of OAL in various disease models. For example, OAL induces increases in blood IFN-γ/IL-12 levels and NK cell counts, and enhances the effects of anticancer drugs (11, 30). OAL also reduces blood IgE levels and improves atopic dermatitis (31). Furthermore, OAL suppresses atherosclerosis by inducing improvement of glucose tolerance and reduction of blood LDL and inflammatory cytokines (12). Importantly, human studies also show that OAL has the protective effect on hyperglycemia and hyperlipidemia (32), the inhibitory effect on bone density loss in middle-aged and elderly women (33), and the effect on improving blood flow (34). Because OAL promotes the phagocytic activity of peritoneal macrophages (41), it is suggested that macrophages are involved in the disease prevention mechanism by OAL.

### Studies on disease exacerbation effect of OAL

On the other hand, there are a few reports that OAL exacerbates pathologies. For example, OAL has been reported to exacerbate collagen-induced arthritis (35) and autoimmune diseases (36). There is also a report that OAL to heat-stressed chickens increases enteritis (37). In addition, although it is not OAL, it has been reported that oral administration of *Porphyromonas gingivalis*, a periodontal disease bacterium, invades the blood from the periodontal lesion site, induces endotoxemia, and exacerbates Alzheimer’s disease (42, 43). In these reports of disease exacerbation by OAL, an increase in blood LPS was observed. In contrast, LPS was not detected in blood in OAL under normal conditions (14). Therefore, under physiological conditions, OAL is hypothesized to act on the immune system of the oral or upper gastrointestinal mucosa to induce disease-preventing effects without inducing elevation of blood LPS concentration (44). On the other hand, in exceptionally severe leaky gut and mucosal immune barrier breakdown due to periodontal disease or immunodeficiency, LPS may be absorbed into blood and cause inflammation. Thus, in the future, it is important to identify complications and medical history that require attention in OAL treatment.

### Diversity of LPS activity depending on bacterial origin

Although there is diversity in the LPS structure depending on the bacterial origin, the differences in the LPS activation mechanisms are less clear. However, at least it is known that lipid A structure influences the difference in LPS activity. For example, LPS from *Bacteroides dorei*, a predominant Gram-negative bacterium in the human gut flora, is not only non-immunogenic *per se*, but also inhibits TLR4-dependent cytokine production (45). The lipid A of *Escherichia*-derived LPS has 6 acyl groups and the lipid A of *Salmonella*-derived LPS has 7 acyl groups, whereas the lipid A of *B. dorei* LPS has 4 acyl groups (46).

On the other hand, lipid A of *P. agglomerans*-derived LPS has 7 acyl groups, similar to *Salmonella*-derived LPS (47). The LD50 of *P. agglomerans* LPS is also almost the same as that of *Escherichia*- and *Salmonella*-derived LPS (48). In addition, *P. agglomeran*s-derived LPS induces pro-inflammatory cytokine production through TLR4 signaling like *Escherichia*-derived LPS and *Salmonella*-derived LPS (49). Therefore, *P. agglomerans*-derived LPS is similar to *Escherichia*-derived LPS and *Salmonella*-derived LPS in terms of TLR4 activity and lipid A acyl group structure.

It is also interesting to explore the diversity of LPS activity by bacterial origin. However, considering the previous studies on OAL shown in **Table 1**, it can be expected that both *Escherichia*-derived LPS and *Salmonella*-derived LPS exhibit disease prevention effects equivalent to *P. agglomerans*-derived LPS when administered orally. This is because the contact of LPS with healthy mucous membranes is considered to be more important than the origin of LPS in the mechanism of disease prevention by OAL as described above.

However, what is important here is the fact that at least oral administration of LPS derived from *P. agglomerans*, a food symbiotic bacterium, has been verified for its safety and disease prevention effect. In the future, it is expected that *P. agglomerans*-derived LPS, whose safety has already been confirmed, will be developed for clinical application as the mainstay of dementia preventive drugs. At the same time, in order to establish a more effective dementia prevention method, the diversity of LPS derived from other Gram-negative bacteria and their OAL effects should be elucidated.

### Preventive effect of OAL on dementia *via* neuroprotective microglia

We recently have demonstrated that OAL prevents dementia (13, 14). OAL inhibited amyloid-β deposition and tau phosphorylation by promoting microglial amyloid-β phagocytosis and prevented brain atrophy. On the other hand, microglia depletion by PLX3397 canceled the preventive effect of OAL on dementia. These results indicate that microglia play an important role in the prevention mechanism of dementia by OAL. Furthermore, comprehensive genetic analysis revealed that OAL-transformed microglia (OAL-microglia) highly expressed genes involved in inflammation control, tissue repair, and promotion of phagocytosis, such as CSF1 receptor (CSF1R), IL-10, and heat shock protein (HSP). The gene expression pattern of OAL-microglia was different from that of normal and dementia-induced microglia. These results indicate that OAL prevents dementia by inducing neuroprotective microglia.

CSF1R regulates survival and proliferation of macrophages, and CSF1R signaling contributes to neuroprotective microglia induction (50–54). In addition, peripheral CSF1 levels correlate with CSF1R levels in CNS (55, 56), and peripheral administration of CSF1R ligands ameliorates neurological disorders (57–59). Indeed, our *in vitro* analysis also showed that CSF1R activation of microglia increased the phagocytosis of amyloid-β, promoted IL-10 and HSP gene expression, and exhibited neuroprotective effects (14). CSF1R ligands include CSF1 and IL-34, and CSF1 further has isoforms of membranous CSF1 (m-CSF1) and secretory CSF1 (s-CSF1). We found that OAL promoted m-CSF1 expression on peripheral leukocytes (14). Therefore, it is strongly suggested that OAL contributes to the prevention of dementia by promoting m-CSF1 expression on peripheral leukocytes, activating CSF1R on microglia, and inducing transformation to neuroprotective microglia. As mentioned above, because CSF1 is also reported to be involved in the maintenance of gastrointestinal motility homeostasis by crosstalk between muscularis macrophages and enteric neurons by OAL (22), CSF1R signaling is thought to play an important role in the disease prevention mechanism of OAL.

### OAL-microglia and CSF1R signaling

The choroid plexus functions as a site of crosstalk between CNS cells and the peripheral immune system (60). In the physiological state, peripheral leukocytes communicate with healthy CNS cells in the choroid plexus for homeostasis maintenance without entering the tissue parenchyma. In contrast, in pathological conditions, peripheral leukocytes sense CNS abnormalities such as alarmins in the choroid plexus and prevent neuropathy by crosstalking with CNS cells through neuroprotective molecules. Interestingly, the choroid plexus behaves like an active barrier under certain conditions. Thus, the choroid plexus selectively recruits immune cells into the CNS, facilitating close communication between peripheral leukocytes and CNS cells to suppress neuropathy.

**Figure 1:**
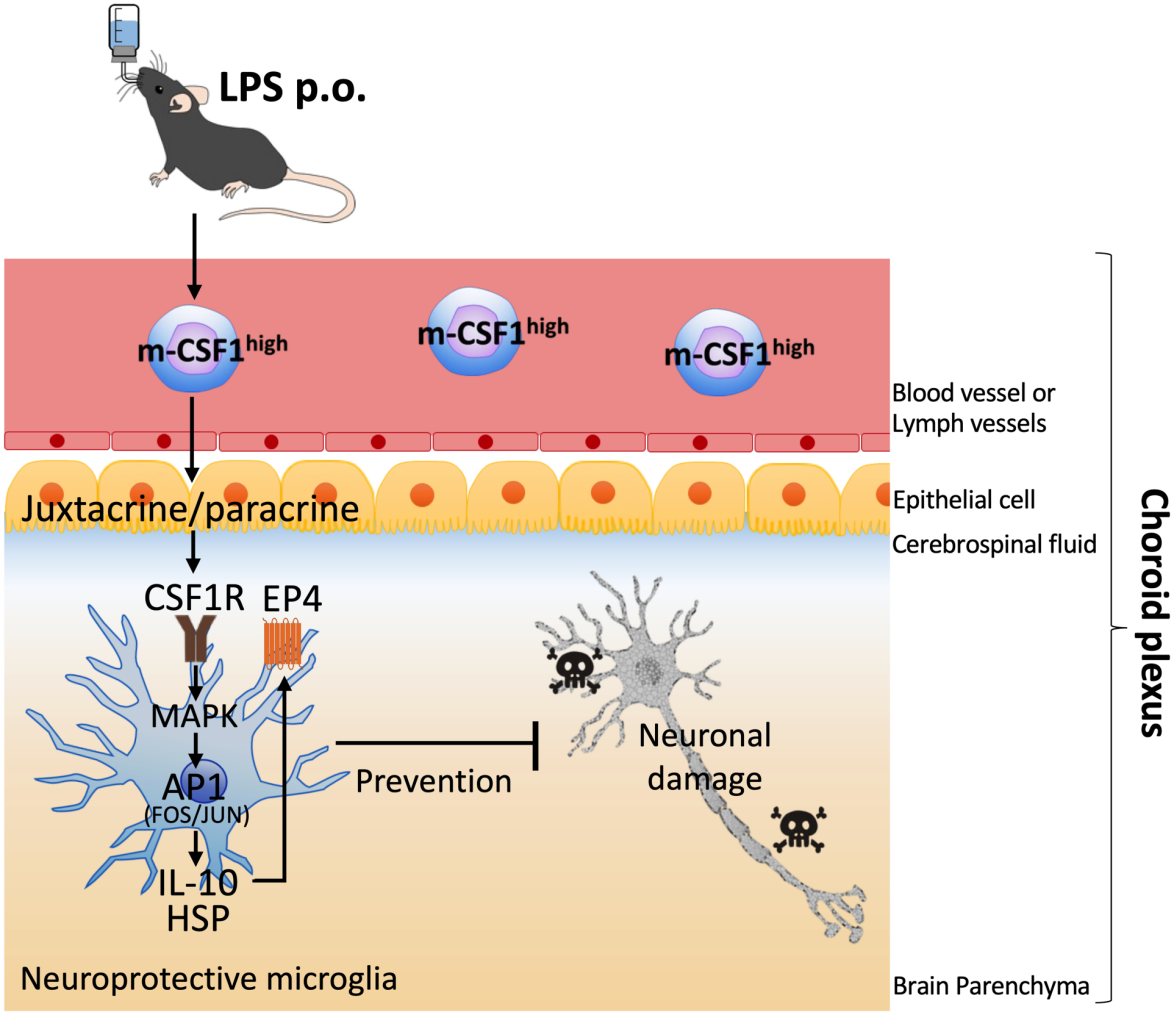
Hypothetical mechanism of induction of neuroprotective microglia by OAL. OAL promotes m-CSF1 expression on peripheral leukocytes. In the physiological state, peripheral leukocytes communicate with healthy CNS cells in the choroid plexus for homeostasis maintenance. In pathological conditions, the microglial CSF1R activation by m-CSF1 on peripheral leukocytes induces transformation to neuroprotective microglia, which contributes to the prevention of dementia. OAL, oral administration of LPS; m-CSF1, membranous colony stimulating factor 1; CNS, central nervous system; CSF1R, CSF1 receptor.

By the way, in contrast to s-CSF1 which has an endocrine effect systemically, m-CSF1 shows local juxtacrine or paracrine effect (61). Major cells in brain parenchyma include microglia, astrocyte, oligodendrocyte, and neuron. Among these cells, only microglia express CSF1R. Therefore, it is considered that OAL-induced m-CSF1 on peripheral leukocytes crosstalk restrictively with microglia *via* CSF1R. Considering the functions of the choroid plexus described above, the choroid plexus is the predicted communication site for restricted signaling between m-CSF1 on peripheral leukocytes and CSF1R on microglia. Indeed, unlike the dementia model, OAL had little effect on expression of the neuroprotective genes in microglia of naïve mice (14). Therefore, only in pathological but not physiological conditions, the microglial CSF1R activation by m-CSF1 on peripheral leukocytes may induce transformation to neuroprotective microglia (**Figure 1**). In such an anomaly-sensing system, m-CSF1 is certainly more rational than s-CSF1, as it transmits signals only to the necessary target in a “just-in-time system” manner. It is likely that OAL exerts its disease-preventing effect in other tissues as well, through mechanisms similar to those in the brain. In other words, it is supposed that OAL-induced m-CSF1^high^ leukocytes monitor the whole body while circulating, and when abnormalities are detected, they activate the CSF1R signal of tissue-resident macrophages, thereby inducing transformation to protective macrophages.

OAL-microglia in dementia models are characterized by high expression of the following genes (14): genes of CSF1R, IL-10, IL-12B, prostaglandin-E2 EP4 receptor (encoded by *Ptger4* gene), c-Jun, and heat shock protein (HSP) family (14). IL-10 is an anti-inflammatory cytokine and interestingly synergizes with IL-12B to regulate inflammation in tumor models (62). Activation of EP4 receptor suppresses brain inflammation (63–65), and also synergizes with CSF1 signaling (66). c-Jun forms activator protein 1 (AP-1), a transcription factor, which is activated downstream of CSF1 signal involving in transformation to anti-inflammatory macrophage (51, 54). AP-1 is also involved in the induction of IL-10, IL-12, HSP (67–70). Exogenous HSP promotes Aβ phagocytosis of microglia and induces neuroprotection (71).

On the other hand, CSF1R activation promotes IL-10 and HSP expression in microglia (14), and MAPK and c-Jun activation are involved in the upstream signals (51, 52, 72, 73). In addition, it has also been reported that EP4 receptor and HSP expression are elevated downstream of IL-10 (74, 75). These facts indicate that the gene expression patterns of CSF1R-stimulated microglia and OAL-microglia are highly similar, suggesting that m-CSF1 and CSF1R are key molecules that mediate neuroprotective OAL-microglia induction. In the future, it is necessary to clarify the detailed mechanism of how CSF1R signaling is involved in the induction of OAL-microglia.

In addition to CSF1R signal, TLR4 is expected to be involved in the OAL mechanism because OAL promotes phagocytosis of peritoneal macrophages in a TLR4-dependent manner (41). However, other OAL mechanisms are still largely unknown. It should be elucidated how CSF1R, TLR4 and other signals are involved in the OAL mechanism.

## Discussion

As one of the efforts to solve dementia, a dietary method called Mediterranean-DASH Intervention for Neurodegenerative Delay diet (MIND diet) has been proposed to prevent dementia (76, 77). The MIND diet includes vegetables and whole grains, and these edible plants contain high levels of LPS (16). Especially in grains, LPS is abundant on the outside because symbiotic bacteria exist on the surface of the plant. Thus, whole grains contain more LPS than refined grains (78). Interestingly, human studies have shown that consumption of brown rice with high LPS content contributes to the prevention of cognitive decline in the elderly (79). Because inactivation of LPS requires exposure to a temperature of 250°C for more than 30 min or 180°C for more than 3 h (80), LPS is hardly inactivated during the cooking process of brown rice. Therefore, it is considered that the activity of LPS does not change whether it is derived from viable or dead bacteria. Accordingly, it is suggested that one of the functional components of MIND diet including brown rice may be LPS.

Brown rice contains as much as 10 μg/g of LPS compared to 0.037 μg/g of LPS in white rice (78, 81). LPS in other cereals is 8.8 μg/g in wheat bran, 7.5 μg/g in wheat germ, and 2.95 μg/g in Barley sprouts (16). One serving of brown rice (65 g) provides 0.65 mg of LPS, and eating three servings of brown rice a day yields 1.95 mg of LPS per day. Assuming a body weight of 60 kg, it corresponds to an LPS intake of 32.5 μg/kg per day. Because LPS is also taken from organic foods other than brown rice, the total LPS intake is expected to be slightly higher than this dose.

As shown in **Table 1**, LPS intake dose in humans that is effective in suppressing blood sugar levels, preventing osteoporosis, and improving blood flow is about 0.5 mg/day, which is 8.3 μg/kg. On the other hand, LPS intake dose in mice that is effective in preventing arteriosclerosis and enhancing anticancer drugs is about 0.3~0.5 mg/kg, which is approximately 60 times higher than the human dose. This difference is probably due to differences in animal species. In mouse dementia model, not 0.3 mg/kg but 1 mg/kg LPS has been shown to prevent cognitive brain decline (13). A simple calculation suggests that the LPS intake dose that can prevent dementia in humans is about 8.3 x 3.3 = 27 μg/kg. In fact, LPS intake was 32.5 μg/kg in the brown rice study that confirmed the dementia preventive effect. Therefore, this assumed capacity can be said to be reasonable. Based on the above, the LPS intake dose that induces dementia-preventing effects in humans is considered to be around 32.5 μg/kg/day. However, assuming application to human clinical therapy in the future, one of the important issues is to strictly determine the optimal dose of OAL in humans.

However, it is difficult to immediately expect the function of OAL only with food because the quality and quantity of orally ingested LPS are uncertain. Although our previous research has revealed that LPS is certainly contained in food, it is still unknown from what source and to what extent LPS is ingested. In addition, plant cultivation using chemical fertilizers and pesticides kills LPS-expressing symbiotic bacteria, thereby reducing the LPS content in food. Therefore, modern humans are considered to have decreased oral LPS intake from diet. Furthermore, because LPS has structural differences such as Lipid A and sugar chains depending on the bacteria (82), it is thought that there is qualitative diversity in the biological activity of LPS. In order to utilize LPS as a functional material, it is necessary to clarify the quality of LPS, standardize the food content, and elucidate the dose-activity relationship regarding preventive effects, thereby optimizing the expression of OAL effects. For practical use, forms such as tablets and powdered supplements are considered desirable.

In addition to the decrease in dietary LPS intake, excessive sterilization, overuse of antibiotics, and a decrease in contact with soil due to asphalt pavement in urban areas further deprives modern humans of opportunities to contact LPS. In other words, it can be said that modern society is suffering from a lack of LPS. Insufficient LPS exposure during childhood suppresses the induction of immune tolerance, and as a result promotes the development of allergic diseases (83), suggesting that LPS plays an important role in establishing the hygiene hypothesis. Furthermore, as described above, because OAL exhibits preventive and therapeutic effects against various diseases, the modern LPS-deficient environment may also be related to the pathological background of diseases other than allergies.

**Figure 2:**
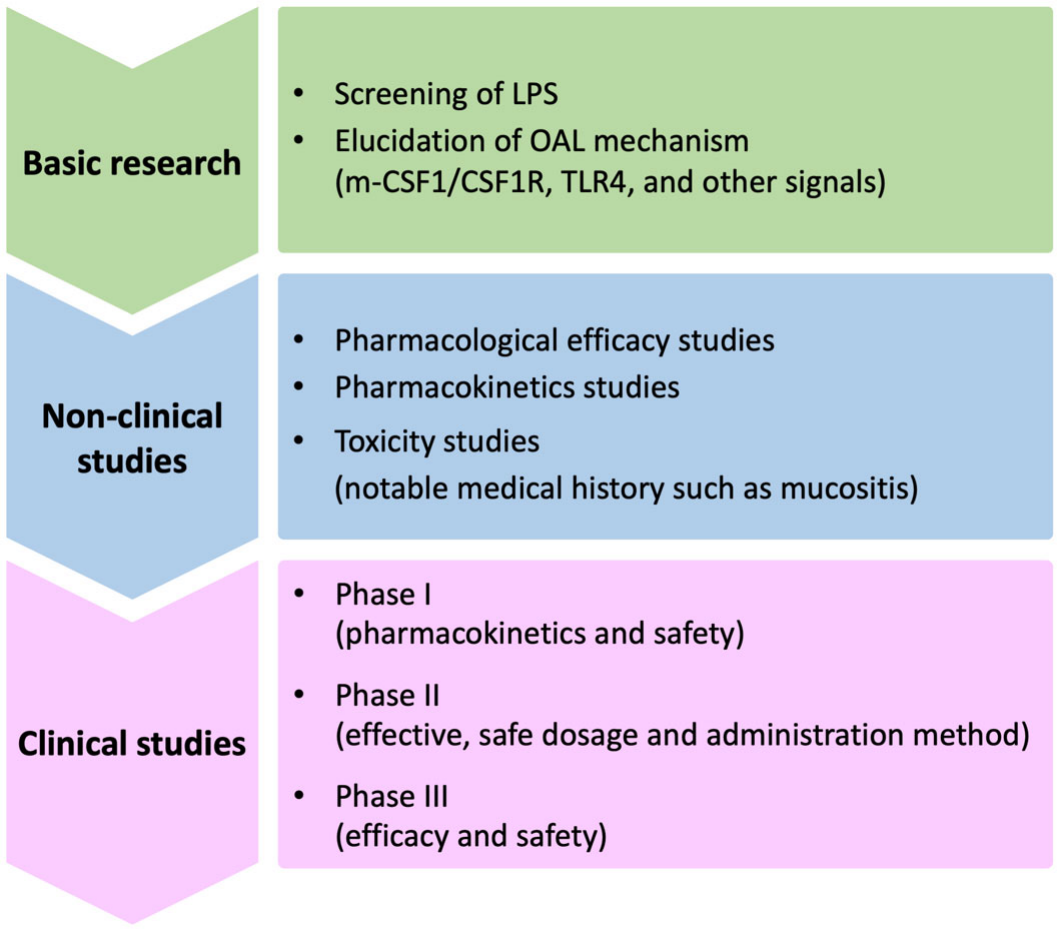
Issues to be solved for clinical application of OAL. Issues to be resolved in the future for clinical application of OAL can be broadly divided into basic research, non-clinical studies, and clinical studies. First, in basic research, the OAL mechanism involving CSF1R signaling must be elucidated. It will also be important to extract LPS with high efficacy through LPS screening. Next, in non-clinical studies, it is necessary to improve the safety and efficacy of OAL through pharmacological, pharmacokinetic, and toxicity studies. Safety in patients with specific and noteworthy medical histories such as mucositis must also be verified there. Based on the above studies, in the final clinical trial, it is necessary to confirm the safety and efficacy in humans and determine the optimal dosage. OAL, oral administration of LPS; m-CSF1, membranous colony stimulating factor 1; CSF1R, CSF1 receptor; TLR4, toll-like receptor 4.

**Figure 3:**
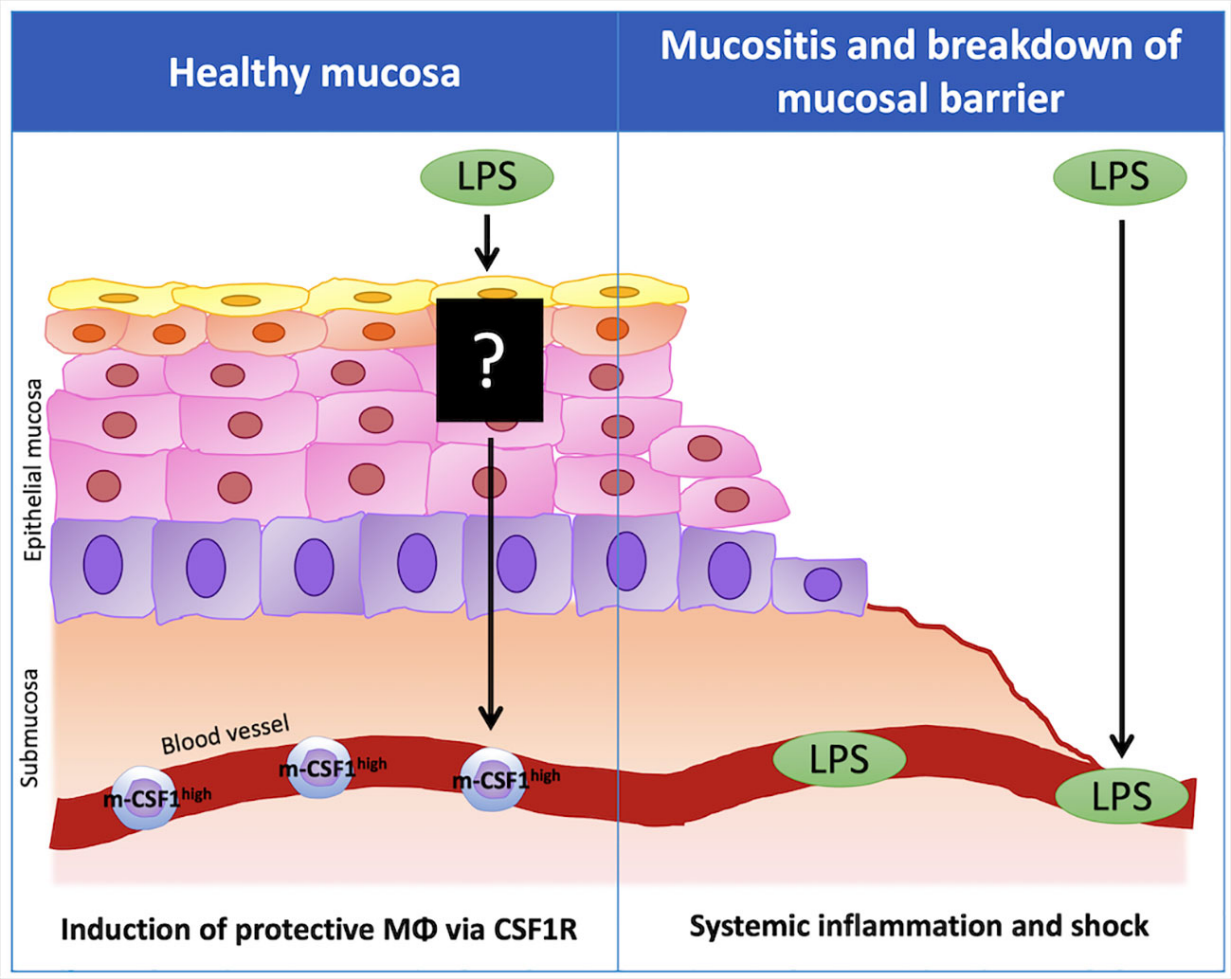
The importance of healthy mucosa for disease-preventing effect of OAL. In healthy mucosa, LPS taken by the oral route does not enter the blood, but acts on the immune system in the oral or upper gastrointestinal mucosa and induces the expression of m-CSF1 in peripheral leukocytes. The leukocytes that highly express m-CSF1 (m-CSF1^high^ leukocytes) patrol the choroid plexus and maintain brain homeostasis. Upon sensing pathological conditions, m-CSF1^high^ leukocytes activate microglial CSF1R signaling in the choroid plexus, inducing transformation to neuroprotective microglia to prevent neuropathy. On the other hand, when the mucosal barrier is disrupted due to mucositis, LPS may be absorbed into the blood and induce inflammation and shock when it circulates systemically. m-CSF1, membranous colony stimulating factor 1; CSF1R, CSF1 receptor.

In order to apply OAL clinically in the future, there are still problems to be solved (**Figure 2**). One of them is the elucidation of mechanism by which OAL exerts its effect. By what route does orally administered LPS induce enhanced m-CSF1 expression in peripheral leukocytes? How do m-CSF1-expressing leukocytes interact with microglia to regulate CSF1R signaling? How do microglia induced by CSF1R activation exert their neuroprotective effects? Do signals other than m-CSF1/CSF1R participate in OAL neuroprotective mechanisms? These questions need to be resolved. Besides, screening LPS with higher efficacy from various LPS from different bacteria origins will further expand the possibilities of OAL. Importantly, as mentioned above, immunodeficient patients with mucosal immune barrier disruption may have to be excluded from OAL treatment in consideration of the risk of LPS absorption into the blood (**Figure 3**). LPS has the risk of inducing inflammation and shock when it circulates systemically. Therefore, in the future, it will be necessary to stipulate prescription conditions for OAL, such as refraining from OAL treatment if patients have periodontal disease or gastrointestinal mucositis. In order to prevent the risk of LPS absorption into the blood, it may be effective to completely cure mucositis before OAL treatment and to take LPS with a mucosal protectant. Based on the fact, it is necessary to investigate the optimal dose of OAL in pharmacological, pharmacokinetic and clinical studies.

In conclusion, this review showed that microglia regulation by OAL could be an innovative preventive method for dementia. One of the advantages of OAL is its simplicity. Because it can be easily taken at home, it is easy to disseminate in society, and it can sufficiently cover the estimated 50 to 100 million people at risk of dementia. The LPS purification method has already been established, and the production cost can be kept low. It is possible to reduce the dementia risk population in a short period by making OAL treatment available to a large number of people. In the future, solving the problems and filling the research gap is expected to lead to the realization of clinical application of OAL.

## Author contributions

HM conceptualized and wrote the manuscript. All authors contributed to the article and approved the submitted version.
